# A Hitchhiker’s guide to CRISPR editing tools in bacteria: CRISPR can help unlock the bacterial world, but technical and regulatory barriers persist

**DOI:** 10.1038/s44319-024-00086-w

**Published:** 2024-02-12

**Authors:** Nicolas Krink, Pablo Iván Nikel, Chase L Beisel

**Affiliations:** 1 The Novo Nordisk Foundation Center for Biosustainability, Technical University of Denmark, 2800 Kongens, Lyngby, Denmark; 2 Helmholtz Institute for RNA-based Infection Research (HIRI), Helmholtz-Centre for Infection Research (HZI), 97080 Würzburg, Germany; 3 Medical Faculty, University of Würzburg, 97080 Würzburg, Germany

**Keywords:** Biotechnology & Synthetic Biology, Methods & Resources

## Abstract

Join us on a journey through the complex and ever-expanding universe of CRISPR approaches for genome editing in bacteria. Discover what is available, current technical challenges, successful implementation of these tools and the regulatory framework around their use.

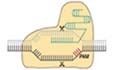

## Don’t panic!

For scientists, engineers, teachers, entrepreneurs, DIY’ers, and other specialists looking to uncover and unlock the secrets of the bacterial world, CRISPR-based tools may jump out as attractive offerings. Numerous studies document their use for editing the genomes of bacteria, from small base swaps to inserting and removing large chromosomal regions. Some of these studies also claim impressive editing efficiencies as well as the ability to create multiple edits in a single attempt. However, deciding where to start can be a daunting task given the diversity of available options and the rapid pace at which new and improved tools are being introduced. With this perspective, our intent is to provide some orientation—and some sense of calm—while navigating the diversity of CRISPR tools, recognizing what needs to be done to make them better and tackling the technical, regulatory and other challenges. Inspired by Douglas Adams’ classic scientific-fiction comedy novel *The Hitchhiker’s Guide to the Galaxy* (1979), this article is our attempt to provide a brief, and hopefully informative, tour with this hitchhiker’s guide to the universe of CRISPR-based editing tools in bacteria.

## Start with the basics

**Figure 1:**
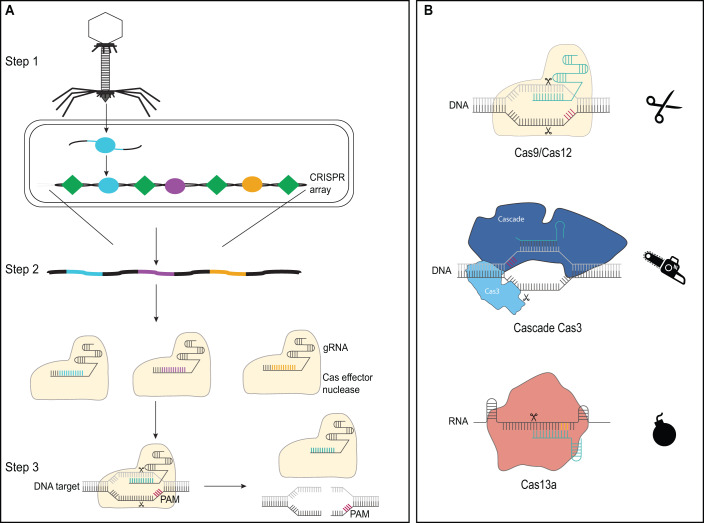
An overview of CRISPR-Cas systems, the source of CRISPR technologies. (**A**) Adaptive immunity by CRISPR-Cas systems encompasses three steps. In the first step (acquisition), a new snippet of an invader’s genetic material is captured and stored as a colored “spacer” in between fixed “repeats” in a CRISPR array. The only key requirement of the spacer sequence is that it is flanked by a protospacer-adjacent motif (PAM) later recognized by the Cas effector nuclease in the third step. In the second step (expression), the CRISPR machinery is expressed, giving rise to a guide RNA bound to the system’s Cas effector nuclease. In the third step, the Cas effector nuclease uses the guide RNA to identify complementary targets in the genome flanked by the PAM; if found, the nuclease is activated, leading to an immune response. In this example, the recognized invader DNA is cut in two, preventing the invader from further replicating. (**B**) Varying immune responses induced by CRISPR nucleases. CRISPR-Cas systems are remarkably diverse, including how they fend off a recognized invader. The examples shown here illustrate three different modes: DNA cutting by Cas nucleases such as Cas9 and Cas12a, DNA degradation by Cas nucleases such as Cas3, and collateral RNA cutting by nucleases such as Cas13. The first two selectively eliminate the invader’s genetic material while the third shuts down the infected cell through widespread degradation of the cell’s transcripts.

To fully grasp CRISPR (Clustered Regularly Interspaced Short Palindromic Repeats) technologies, it is important to understand where they come from. Importantly, these technologies were not invented from scratch but instead derived from bacterial CRISPR-Cas systems, the only known adaptive immune systems in bacteria and archaea (Marraffini, 2015). They operate through three general steps to recognize and eliminate foreign invaders, such as plasmids or bacteriophages (Fig. 1A). In the first step, the systems store an ~30-nucleotide fragment of the invader’s DNA within so-called CRISPR arrays. In the second step, the CRISPR arrays serve as a template to produce guide RNAs (gRNAs) that pair with the system’s Cas effector nuclease, with Cas9 as the best-known example. In the third step, the paired nuclease-gRNA searches within the cell for the same DNA fragment representing the original invader. If the invader is found, the nuclease kicks off an immune response.

“Our intent is to provide some orientation—and some sense of calm—while navigating the diversity of CRISPR tools.”

Recognition of this DNA fragment follows two criteria: base pairing between the DNA fragment and the gRNA, and the presence of a flanking sequence generally called a protospacer-adjacent motif (PAM), which varies widely in sequence and length from one to eight bases. The form of the immune response depends on the nuclease (Fig. 1B): the nucleases Cas9 and Cas12a cut recognized DNA to halt an infection, Cas3 degrades one-half of the recognized DNA, while Cas13 begins non-specifically cutting RNA, causing the cell to shut down. These examples represent only a fraction of the natural diversity of CRISPR-Cas systems, with each new layer ripe for use in applications spanning genome editing, tailored-spectrum antibiotics, molecular point-of-care diagnostics, and more (Pickar-Oliver and Gersbach, 2019).

“To fully grasp CRISPR technologies, it is important to understand where they come from.”

**Figure 2:**
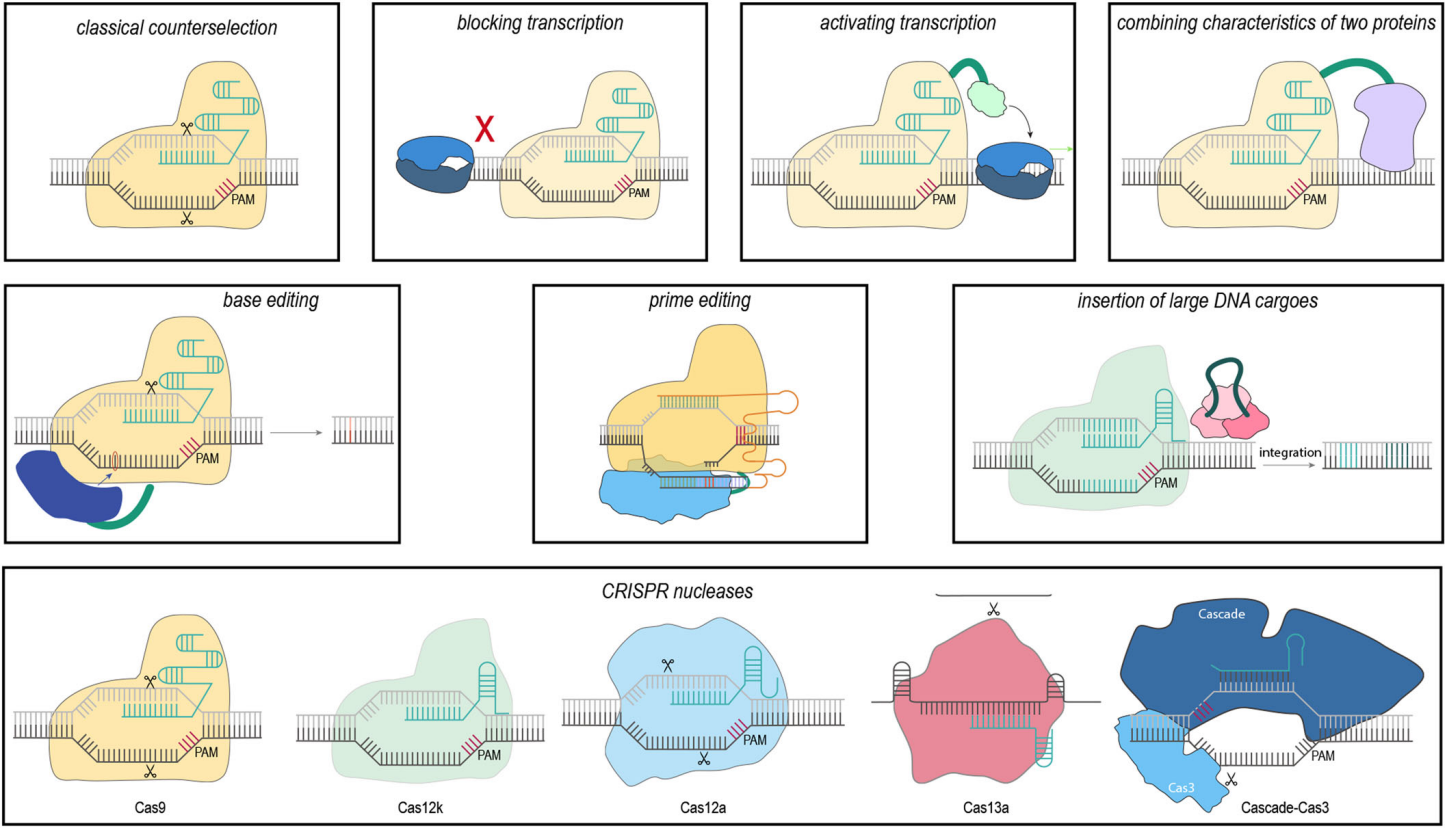
A diversity of CRISPR-based technologies for bacterial genome editing. The current list of available editing technologies will likely continue to expand. These technologies include more traditional approaches such as selectively cutting unedited DNA to enrich for edited cells to activating or repressing expression of selected genes. Recent approaches have enabled CRISPR-driven-precision editing using base editors, prime editors, and CRISPR transposons. A myriad of distinct Cas effector nucleases is also available for these editing approaches, from the original Cas9 nuclease to more recently discovered nucleases such as Cas13a or Cas12k (part of CRISPR transposons).

The ability to design and express multiple gRNAs, as well as the availability of single-protein nucleases such as Cas9, drove the development of CRISPR-based tools as technologies. Bacterial genome engineering has been a particularly busy area despite the strong focus on eukaryotic applications (Volke et al, 2023), with a wide range of technologies now available (Fig. 2). Traditionally, a DNA-targeting Cas nuclease such as Cas9 is coupled with recombineering techniques—for instance, λ-Red—to introduce a range of genomic edits. Recombineering generates an edit, while the Cas nuclease is directed to cut only the unedited sequence. Because bacteria normally succumb to DNA breaks caused by Cas nucleases, cells that underwent editing are spared and can expand. Alternatively, Cas nucleases engineered with mutations that fully disrupt DNA cutting, called catalytically-dead Cas nucleases, can be used to block transcription of target loci, a process termed CRISPR interference (CRISPRi). These same nucleases can also be engineered to recruit transcription factors to up-regulate transcription, a process termed CRISPR activation (CRISPRa). Finally, the nucleases can be combined with an error-prone DNA polymerase that facilitates introduction of random mutations downstream of the target, a technology termed EvolvR.

Cas nucleases have also been mutated to cut only one of the two DNA strands to generate a DNA nick, which have also proven invaluable when fused to different enzymatic domains. As part of one technology called base editing, the nicking Cas nuclease is fused to an enzymatic domain that deaminates a DNA base. Deamination switches the identity of the modified base—C to U, A to I—which is then hard-coded as a standard DNA base. The end result is that individual bases within the target are switched.

Separately, another technology called prime editing combines the nicking Cas nuclease with a reverse transcriptase and a 3′-extended gRNA. The nicked DNA base-pairs with the gRNA extension, and the reverse transcriptase extends the DNA based on the gRNA, resulting in a newly synthesized sequence. This sequence can be integrated through DNA repair into the target DNA. More recently, CRISPR-associated transposons, mobile genetic elements that interweave CRISPR-based tools and transposons, were discovered and quickly adopted for programmable DNA insertion. DNA insertion takes place ~50 bases downstream of the target site, and DNA cargoes as long as 10 kilobases have been successfully introduced. Finally, the ability of Cas3 nucleases to degrade large stretches of DNA has been used to generate large deletions spanning up to 400 kilobases (Csörgő et al, 2020).

These capabilities have given rise to a wide range of options for genome editing in bacteria. Most have been applied to model bacteria such as *Escherichia coli*, although they are being increasingly applied to non-model bacteria. Given the plethora of options, the trick is figuring out where to start.

## Search for an answer, be it a specific CRISPR tool or the number 42

CRISPR-based tools are not the universal answer to bacterial engineering—let alone to all of life’s mysteries. However, they can be incredibly useful in the right context. To get there, we recommend beginning with identifying the goal and recognizing that multiple options are available but also come with distinct tradeoffs. If the intent is to eliminate an expressed gene product—that is, an essential gene that cannot be deleted—CRISPRi has been the most reliable and widely used tool. Expressing a catalytically-dead Cas nuclease that targets DNA along with a gRNA represents the simplest approach to implement. In addition, predictive design tools based on machine learning are becoming available, which can maximize silencing and aid in the design of multiplexed silencing or high-throughput screening. However, the resulting gene silencing rarely achieves 100%. Gene silencing approaches based on RNA-targeting Cas nucleases are also becoming available as complementary strategies. If a true knockout is desired, base editing is proving a useful means to introduce pre-mature stop codons. The caveat is that the deaminase activity can extend throughout the genome, leading to background mutations often ignored in bacterial editing efforts.

“CRISPR-based tools are not the universal answer to bacterial engineering—let alone to all of life’s mysteries. However, they can be incredibly useful in the right context.”

If the intent is to introduce random mutations over a stretch of DNA, for instance, for directed evolution, EvolvR appears to be the best option—albeit with the limitation that it has only been applied in one bacterium: *E. coli*. Base-editing has also been applied to mutagenize genes, but a much larger set of gRNAs are needed, and PAM requirements and the types of base conversions that can be achieved impact how extensive the mutagenesis can be. For precise edits, base editing is rarely useful. At first glance, prime editing is more attractive owing to the greater flexibility in introduced edits, although it so far underperforms even in *E. coli*. Here, traditional CRISPR-based counterselection remains the best approach, even if editing efficiencies can vary widely.

For large insertions, CRISPR-based transposons have proven incredibly effective in multiple bacteria; the caveats are that insertion comes with ~100-bp transposon ends that remain as editing scars, and the multi-gene transposons combined with the DNA cargo lend to very large expression constructs. Finally, for gene activation, CRISPRa is starting to show promise, even if activating any of gene-of-interest remains out of reach. Instead, a promoter can be inserted via traditional CRISPR editing or with CRISPR transposons.

## As useful as a towel, but ample room for improvement

While CRISPR-based tools have much to offer for bacterial editing, there is still ample room for improvement and important bottlenecks remain. The first—but often overlooked—obstacle is laying the groundwork to even express the CRISPR machinery in the bacterium of interest. Such groundwork includes the ability to efficiently introduce foreign DNA into the bacterium—via electroporation, conjugation or phage delivery—circumvent any number of strain-specific immune defenses, faithfully replicate the DNA, and ensure sufficient transcription and translation of the CRISPR machinery (Vento et al, 2019). Reaching this stage remains non-trivial to say the least, particularly when venturing into non-model microbes. It also normally requires culturing the microbe under laboratory conditions, which itself is not possible for most microbes in the world. Base-editing of microbes in their natural habitat, a process called in situ editing, could provide access to bacteria normally off-limits in laboratory settings, as well as afford opportunities to manipulate existing microbiomes for different applications. However, this approach presents its own unique challenges, particularly regarding CRISPR delivery and efficient editing without perturbing the microbial population.

Even when CRISPR-based tools can be reliably introduced and expressed, jump-starting even existing tools in a new bacterium can be difficult and unpredictable to say the least (Waller et al, 2017). For instance, the expression of some Cas proteins can be cytotoxic, even in the absence of genome targeting. Moreover, the efficacy of recombineering coupled with CRISPR-based counterselection varies greatly even between related strains. One general workaround is attenuating targeting activity, such as by introducing mutations into the guide sequence or using a less-active Cas nuclease; this approach can paradoxically improve editing even in the absence of a recombinase by promoting DNA repair through the host’s machinery (Collias et al, 2023). However, it remains unpredictable and requires screening of attenuation approaches. Overall, these challenges are further exacerbated when trying to push the limits of the technology, whether performing large-scale multiplexing or massively high-throughput genetic screens or making exceedingly large changes to the genome.

“Even when CRISPR-based tools can be reliably introduced and expressed, jump-starting even existing tools in a new bacterium can be difficult and unpredictable….”

We list these challenges not to discourage, but instead to lay out a path forward and set some expectations about the required effort. Consider this a call-to-arms to further invest in interrogating the natural diversity of bacteria and working toward standardized pipelines for onboarding CRISPR tools. Continued efforts to mine the natural diversity of Cas proteins as well as engineer them for our own needs will help to expand the available toolbox. Until then—and possibly even then—some amount of screening of approaches, Cas protein homologs and engineered variants, and gRNAs and target sites will be necessary to identify the right combination for your application. We promise that the effort is well worth it, and the lessons learned along the way will be invaluable to the community at large.

## Don’t turn into a penguin—and move toward applications

**Figure 3:**
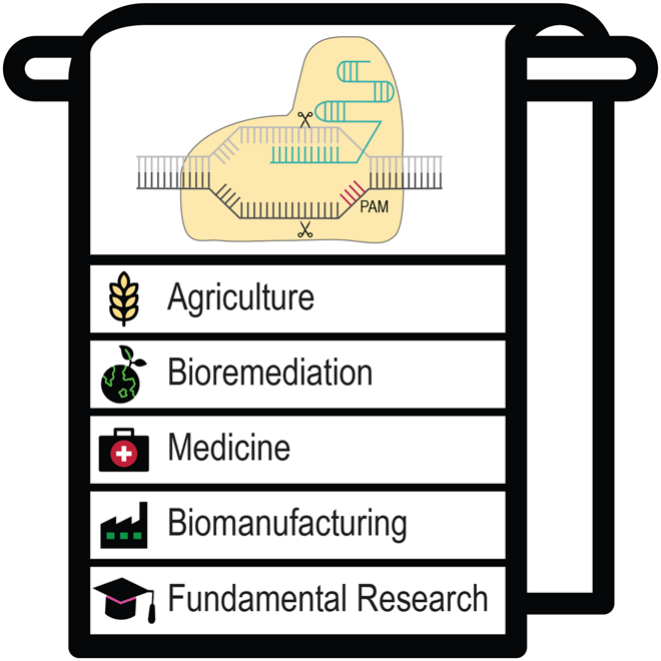
Application areas of CRISPR-based bacterial genome editing. Like a towel in the Hitchhiker’s guide, many applications fall into the broad categories of agriculture, bioremediation, cell-based therapies, cell-based diagnostics, biomanufacturing, and fundamental research.

While further advancing CRISPR-based tools across bacteria is a major undertaking, there are pressing needs that the existing and future tools could begin addressing. For a start, there is an immense opportunity to continue probing the genetics and physiology of the microbial world. Only a fraction of these microbes has been genetically manipulated, and the implementation of CRISPR-based tools could help spark a revolution in microbial studies and our ensuing understanding. Apart from advancing fundamental knowledge, CRISPR-based editing approaches could be immediately and directly applied in bacteria to tackle a diverse array of societal, environmental and economic needs (Fig. 3).

A major opportunity exists within industrial biotechnology working towards a sustainable and circular bio-based economy (Han et al, 2023). Engineered microbes have long held the potential to be converted into microbial chemical factories to enable a move away from fossil fuels. Using non-biodegradable waste, such as plastics, could become a new generation of cheap feedstocks that concomitantly reduce waste. Bioremediation will also become increasingly important to remove pollutants and contaminants. In agriculture, plant-associated microbes already play an important role in crop development and health; engineering these same microbes could further bolster yields and improve growing crops in the face of climate change, as well as reduce the use of fertilizers.

In medicine, CRISPR-edited microbes can offer a distinct form of cell-based therapies to address conditions ranging from cancer to inborn metabolic diseases. Bacteria naturally found in a given microbiome, such as the digestive tract, can be engineered in the laboratory and administered like a probiotic. As coaxing these bacteria to take up residence in the microbiome can be challenging, an alternative approach is editing existing bacteria already present in the human body in situ—an emerging field of microbial engineering. Finally, biocomputing with engineered microbes, such as using bacteria to translate environmental cues into defined outputs such as forming biofilms, producing antibiotics, or generating electricity, remains a less-developed area that could greatly benefit from the ability to construct scalable CRISPR-based regulatory circuits.

The full suite of CRISPR-based tools will likely be needed for these advances, whether for optimizing strains through removal of competing pathways or cellular responses, inserting heterologous metabolic pathways, or rewiring signaling pathways. Advancing in any of these areas will also require not only moving beyond the few model bacteria but also developing in situ editing as an area of research unto itself. We also see a tremendous opportunity to integrate high-throughput approaches and machine learning to accelerate CRISPR design and implementation (Kolasinliler et al, 2023). Finally, we see ample synergy between basic microbial discovery, CRISPR tool development, and application development that build onto each other. Overall, the application space for CRISPR-based bacterial genome engineering technologies is incredibly broad, from biotechnological to medical and environmental areas of use. Over the past decade, we have seen several proof-of-principle examples, but we are convinced that this has been just the beginning.

## Dealing with Vogons: legislation around CRISPR technologies

Technologies serve as tools that can potentially benefit humanity, but they can also be misused, whether intentionally or inadvertently. This is particularly applicable to molecular biology tools such as CRISPR-based genome editing, that can be broadly implemented to make diverse types of edits in living matter. For instance, CRISPR-based editing has generated major discussions about human germline editing and gene drives for pest management.

In Europe, the usage of emerging technologies is governed by the precautionary principle as the cornerstone of the technological risk assessment framework. This principle ensures that the impacts of novel technologies, including those associated with CRISPR-based genome editing, are evaluated with the utmost regard for environmental, human and general safety.

Following a rather general trend, the legislation focuses on using CRISPR-based genome editing tools for eukaryotic organisms, mainly plant and mammalian cell editing. Unfortunately, these same provisions are being applied uniformly across all kingdoms of life. With the decision of the European Court of Justice in 2018, which is currently under revision, microorganisms with genomes edited using CRISPR—categorized as a new breeding technology—are considered GMOs, regardless of whether the sequence alterations could have occurred naturally (Hjort et al, 2021). In fact, the concept of transgenic elements in bacteria can be questioned overall, considering the fairly fluent genetic transfer that accrues during bacterial evolution in the environment.

As indicated above, CRISPR methods can be implemented to introduce point mutations that either change or abolish functions of enzymes and proteins, engineer small and large knockouts or even integrate large *cis*- or *trans*-genetic elements. They all have the commonality that these events could theoretically have happened naturally and are, after the process, nearly impossible to differentiate from natural genetic modifications.

Compared to the EU, the US government differentiates between *cis*- and *trans*-genetic elements for the definition of GMOs (Marden et al, 2023). As long as foreign DNA is not introduced into the organism, the organism is not classified as a GMO. This definition, following previous arguments around what foreign DNA means in microorganisms, holds great potential for changing the specificity of a natural enzyme to function similarly to a heterologous one via genome edits. In China, genome editing regulations, especially for the use in humans, have been tightened, after previous controversial research. For non-mammalian organisms, the regulation is progressive but remains case-by-case dependent. Similar to the USA and the EU, specific regulations for microorganisms, especially bacteria, are not in place, which can remain challenging but also provides opportunities to better align regulations with existing capabilities (Gao et al, 2018).

“…it is necessary to have progressive, science-based legislation for genome editing especially in microorganisms…”

In all cases, justification and case-based arguments are still required to obtain the approval of the relevant authorities. We see the current legislation, especially in the EU, as an impediment to the green transition to the future bioeconomy and the application spectrum that can be pursued. We want to stress that it is necessary to have progressive, science-based legislation for genome editing especially in microorganisms that allows the bioeconomy to utilize the technology’s full potential while keeping a balance between technological advances and the need for safety to prevent harm to human or planetary health.

“…we need to institutionalize and structure global efforts to develop and implement CRISPR-based editing approaches, including benchmarking in different bacterial hosts.”

## So long and thanks for all the CRISPR

Despite the technical and societal challenges highlighted in this article, CRISPR-based bacterial genome engineering continues to revolutionize microbial sciences. This broad impact cannot and, in our opinion, should not, be underestimated and has disruptive potential for several applications, ranging from fundamental research to cell factory design, medical applications and in situ environmental engineering. However, we need to institutionalize and structure global efforts to develop and implement CRISPR-based editing approaches, including benchmarking in different bacterial hosts.

We have not found the silver bullet of a universal Cas-based genome engineering technology that functions in an organism-agnostic fashion. Even if that might be impossible, we contend that this should be a goal and ambition, especially now that we have a suite of synthetic biology tools that facilitate genome manipulation. For future applications such as in situ genome engineering of microbiomes, efficient broad and targeted delivery systems need to be developed and made widely available. Overall, the focus on the development of CRISPR-based prokaryotic genome engineering technologies should not be lost. The mid- and long-term impacts of improved Cas-based genome engineering technologies for microorganisms on our future societies cannot be overestimated.

“The mid-and long-term impacts of improved Cas-based genome engineering technologies for microorganisms on our future societies cannot be overestimated.”

### Supplementary information

Peer Review File
