# Supplementary material for: A Hitchhiker’s guide to CRISPR editing tools in bacteria: CRISPR can help unlock the bacterial world, but technical and regulatory barriers persist
Source: EMBO Rep. 2024 Feb 12;25(4):1694–9. doi: 10.1038/s44319-024-00086-w (PMC11014848; doi:10.1038/s44319-024-00086-w)
Supplement: Supplementary file 1 — Peer Review File [file 44319_2024_86_MOESM1_ESM.pdf]

EMBO reports

A hitchhiker's guide to CRISPR editing tools in bacteria

Nicolas Krink, Pablo Nikel, and Chase Beisel

Corresponding author(s): Chase Beisel (chase.beisel@helmholtz-hiri.de)

Review Timeline:

Submission Date:	23rd Jan 24
Accepted:	30th Jan 24

Editor: Holger Breithaupt

Transaction Report: This manuscript was editorially reviewed.
